# Morphological diagnosis of hematologic malignancy using feature fusion-based deep convolutional neural network

**DOI:** 10.1038/s41598-023-44210-7

**Published:** 2023-10-09

**Authors:** D. P. Yadav, Deepak Kumar, Anand Singh Jalal, Ankit Kumar, Kamred Udham Singh, Mohd Asif Shah

**Affiliations:** 1 Department of Computer Engineering and Applications, G.L.A. University, Mathura, 281406 India; 2 Department of Computer Science, NIT Meghalaya, Shillong, 793003 India; 3 School of Computing, Graphic Era Hill University, Dehradun, 248002 India; 4 Kebri Dehar University, Kebri Dehar, Ethiopia; 5 Woxsen University, Kamkole, Sadasivpet, Hyderabad, Telangana 502345, India; 6 Division of Research and Development, Lovely Professional University, Phagwara, Punjab 144001, India; 7 Research Fellow, INTI International University, Persiaran Perdana BBN Putra, Nilai, Negeri Sembilan, 71800, Malaysia

**Keywords:** Energy science and technology, Engineering

## Abstract

Leukemia is a cancer of white blood cells characterized by immature lymphocytes. Due to blood cancer, many people die every year. Hence, the early detection of these blast cells is necessary for avoiding blood cancer. A novel deep convolutional neural network (CNN) 3SNet that has depth-wise convolution blocks to reduce the computation costs has been developed to aid the diagnosis of leukemia cells. The proposed method includes three inputs to the deep CNN model. These inputs are grayscale and their corresponding histogram of gradient (HOG) and local binary pattern (LBP) images. The HOG image finds the local shape, and the LBP image describes the leukaemia cell's texture pattern. The suggested model was trained and tested with images from the AML-Cytomorphology_LMU dataset. The mean average precision (MAP) for the cell with less than 100 images in the dataset was 84%, whereas for cells with more than 100 images in the dataset was 93.83%. In addition, the ROC curve area for these cells is more than 98%. This confirmed proposed model could be an adjunct tool to provide a second opinion to a doctor.

## Introduction

Bone marrow, a soft and versatile tissue accessible in bone depressions, is the site of hematopoiesis, producing millions of blood cells every day^1^. Hematopoiesis promotes the formation of blood, which is one the essential components of the human body and it is composed of 80 percent water and 20 percent solid^2^. The red blood cells (RBC), white blood cells (WBC), platelets and plasma are the four blood components available^3^. White blood cells make up roughly 1% of blood. e. 1 WBC is present in every 100 red blood cells. The neutrophils, lymphocytes, eosinophils, basophils and monocytes. These cells have an average count of 60%, 30%, 5% and, 4 %, under 1% of the total WBC count, respectively^4^. Blood cell cancer refers to bone marrow contains leukemia cells, which are abnormal WBC^5^.

The current prognosis for leukemia is not encouraging, and the disease continues to pose a significant risk to the health of humans. Leukemia was estimated to be the 15th most common cause of cancer incidence and the 11th most common cause of cancer-related mortality worldwide in 2020. It was responsible for 474,519 cancer-incident cases and 311,594 cancer-related deaths. In addition, leukemia is the most common cancer in children younger than five. It is also responsible for the highest percentage of deaths, which substantially costs individuals, families, and countries^6^.

Acute lymphoblastic leukemia (ALL), acute myeloid leukemia (AML), chronic lymphocytic leukemia (CLL), and chronic myeloid leukemia (CML) are the most common types of leukemia identified^7^. The rapid deterioration of the patient is caused by acute leukemia, while chronic leukemia is characterized by gradual progression and may be lymphocytic or myelogenous. Two methods are widely used to diagnose leukemia: The French-American-British (FAB) classification and the World Health Organization (WHO) proposal.

Early identification of this disease is critical for successful treatment. Pathological testing, full blood count, aspiration biopsy, and bone marrow aspiration involving the creation of microscopic blood smear images taken from the potential patient are the methods used to diagnose leukaemia^8^. The leukaemia laboratory test is time-consuming and inconvenient, requiring extra time and effort^9^. Manual analysis for leukaemia diagnosis can result in diagnostic variability and inaccuracies in blast cell counting. As a result, there may be discrepancies in diagnostic outcomes^10^. The significant challenges with manual leukaemia diagnosis are non-standardization, conflicting, and subjective findings due to the possibility of human error or differing expert opinions^11^.

The morphological features-based study of the blood cells is less accurate than automated techniques^12^. When an extensive dataset is available, a machine learning (ML) algorithm can help differentiate the blood cells with leukemia from the healthy cells.

Various studies have proved that Machine learning (ML) techniques are more helpful in detecting blast cells from healthy cells and are gaining popularity, as it is faster and more accurate than traditional diagnosis methods^13^. It can be formulated as an image classification task because the cytomorphological analysis is focused on evaluating microscopic cell pictures^14^. In the field of natural image and visual question-answering classification, deep convolution neural networks (CNNs) have proven very effective^15,16^. CNNs have recently been successfully applied to different medical imaging activities, including the identification of skin cancer^17–19^, the assessment of retinal disorders^20^, and the analysis of histological sections^21,22^, e.g. by mitosis detection^23^, the detection and analysis region^24^ or the segmentation of tissue types^25^. This propels us to apply CNNs to the cytomorphological characterization of platelets, specifically those significant in AML. Past work on leukocyte order has predominantly been centered on feature extraction from cytological images^26,27^.

More focus was given to lymphoblastic leukemia, where the cytomorphology is less diverse than in the myeloid case^28,29^. In medical image analysis, supplying sufficient numbers of labelled images for deep learning models to work has proven to be challenging due to restrictions on the availability and the cost of expert time to provide ground truth annotations^30,31^. Therefore, numerous research focused on data sets restricted by the number of patients included or the classification of individual cytological images^32,33^. So far, applications of CNNs to classify white blood cells have concentrated on differentiating subtypes such as erythroid and myeloid precursors^32^.

Matek et al.^34^ have used the ResNext model to classify leukemia cells. They improved the dataset size using augmentation techniques. The augmented dataset contains 15000 images, which took approximately 96 h to train and test the model. In addition, the method's sensitivity toward the cells having less number of images in the dataset is less. Boldu et al.^35^ proposed ALNet by coming to the two modules from VGG16 and VGG19. VGG16 module performs the classification of 4 class, and vgg16 perform the classification of 2 class. They reported classification accuracy of 92% on cells and 100% on smears. Eckardt et al.^36^ use a multi-step deep CNN model using the transfer-learning technique to segment and classify bone marrow cells. Their method classifies bone AML and healthy control with an accuracy of 87%. Khandekar et al.^37^ applied the You Only Look Once (YOLOv4) deep CNN model to classify blood smears. They perform preprocessing to resize the image and maintain orientation. After that, the concerned object of interest is detected using the segmentation technique, and finally, the feature is extracted using a deep CNN model. Their method reported an F1-score of 92% and a recall of 96%. The rest of the recent methods have been summarized in Table 1

**Table 1:** Summary of the recent work using machine learning and deep learning.

Author	Method	Dataset Size	Accuracy (%)
Talaat et al.^38^	OCNN	30,000 images	94.04
Rahman et al.^39^	SVM, CNN, Alex-net model	260 images	98.11
Ansari et al.^40^	Generative adversarial network (GAN)	938 Images	99
Safuan et al.^41^	CNN, VGG, Alexnet, and GoogleNe	1800 Images	99.13
Pallegama et al.^42^	CNN	841 Images	98.53
Rahman et al.^43^	ResNet50 CNN	3262 images	99.84
Revanda et al.^44^	Mask R-CNN	301 multi-cell image	83.72
Sorayya et al.^45^	ResNet-50, VGG-16 CNN	12,528 images	81.63, 84.62, 82.10
Mallick et al.^46^	Five-layer DNN classifier	72 samples with 7128 genes	98.2
Ahmad et al.^47^	Convolutional generative adversarial network	6562 images	99
A. Batool et al.^48^	Lightweight efficientnet-B3	15,114 images	99.31
Rejula et al.^49^	Adaptive neuro-fuzzy neural network	12,500 images	97.14
Elhassan et al.^50^	Deep convolutional autoencoder (DCAE)	18,365 images	97
Ahmad et al.^51^	DenseNet121-ResNet50-MobileNet	10,661 images	98.2

In short, all these methods have a high potential for classifying blood smears. However, the blood smears having less number of images in the dataset need to be explored for better classification. The leukemia cell's morphological characteristics are very similar, which makes it difficult to differentiate them. In addition, a key challenge is cells having less than 100 images in the dataset, which needs a highly sensitive model for identification. Therefore, in the proposed approach, we developed a multilevel feature fusion-based 3SNet for the leukemia cell classification.

The paper's significant contribution is as follows.

1. We introduced 3SNet, a novel multi-scale feature fusion-based deep learning model with depth-wise convolution blocks that efficiently differentiate leukemia cells using less computational resources.
2. The fewer images of the leukemia cell in the dataset and morphologically similar characteristics make the problem more challenging. Hence, Leukemia cell image and their corresponding LBP and HOG images at three scales are used to extract spatial features, and the fusion technique generates an enhanced feature pool. That makes the system more sensitive toward leukemia cells having fewer images in the dataset.
3. We experimentally demonstrated that the proposed model outperforms the AML-Cytomorphology_LMU dataset.

The rest of the paper is organized as follows.

In "Proposed method" section, the proposed method algorithm and model architecture have been elaborated. The result of the 3SNet is discussed in "Results" section, whereas in "Discussion" section, a comparison of the results with the state-of-the-art method has been discussed. Finally, in "Conclusion" section, we have concluded the proposed method.

## Proposed method

**Figure1:**
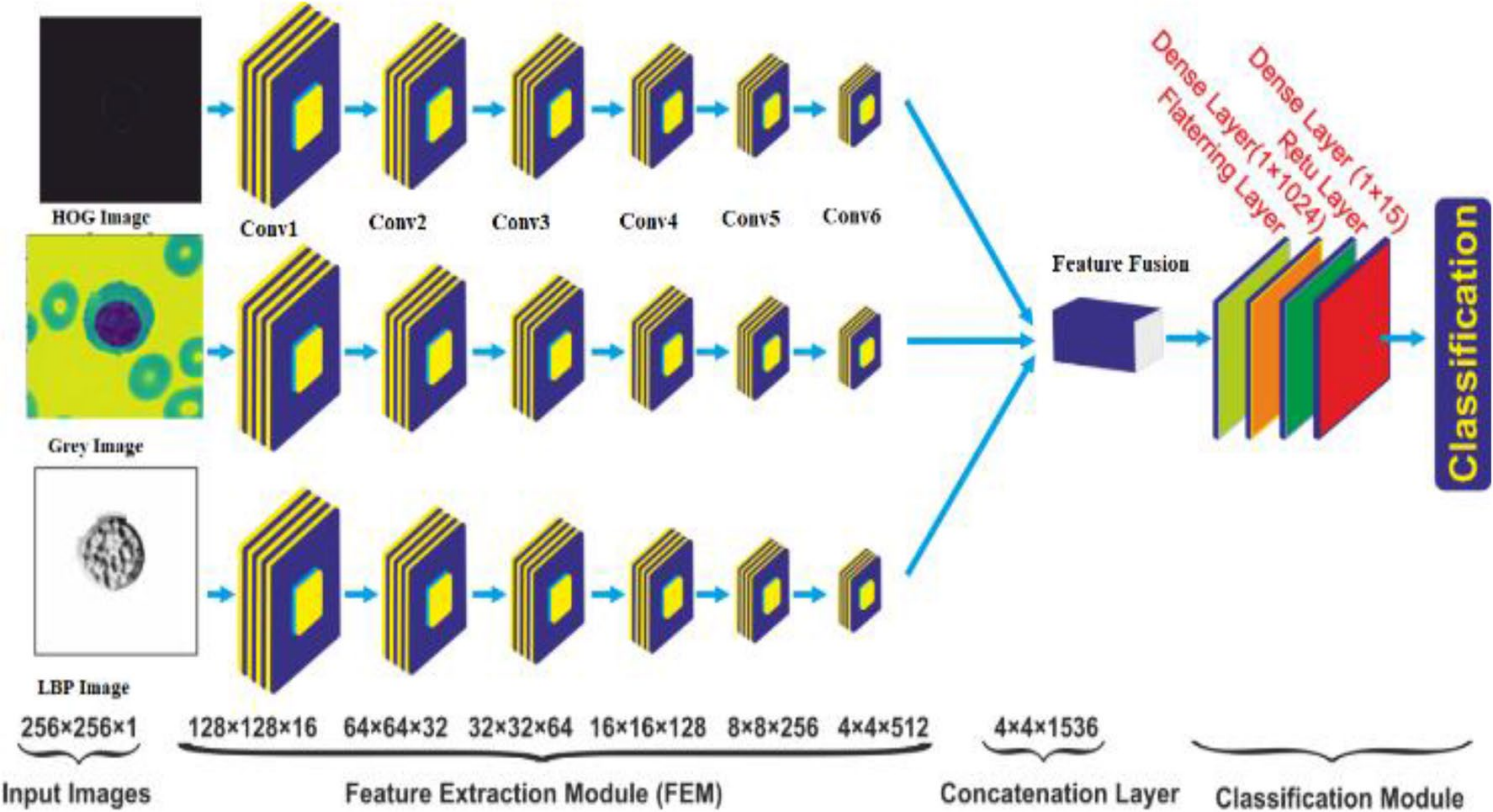
Proposed 3SNet for the leukemia diagnosis.

In this study, we developed a deep convolutional neural network model called 3SNet, which incorporates a multilayer feature fusion approach. The architecture of 3SNet is depicted in Fig. 1. The feature fusion model employed in this study is designed to extract features from the grey image as well as the corresponding histogram of oriented gradients (HOG) and local binary patterns (LBP) images. Subsequently, the aforementioned features are integrated in order enhance their effectiveness, after that the classification module is added to performs classification leaukemia cells.

The convolution blocks are designed using depth-wise convolution techniques to reduce the computation costs. Several methods in the past have done a significant job of improving leukaemia cell classification. However, several limitations of these methods motivated us to design a robust and efficient model. A detailed summary of the models is described in Table 2.

**Table 2:** The detailed summary of the previous models used for leukemia classification.

Deep CNN	Neurons	Limitations
VGG16	33 × 10^6^	This model is slow in training and computationally expensive due to many trainable parameters
AlexNet	24 × 106	Due to the large number of trainable neurons, AlexNet is also costly. Moreover, the model is unable to detect all high-dimensional spatial features
ResNetXt	23 × 10^6^	ResNeXt is a fifty-layer deep CNN model that can extract high-dimensional features that require a large training dataset. In addition, it cannot be used for real-time applications due to the significant number of trainable parameters
DenseNet-121	7.2 × 10^6^	DenseNet-121 has significantly less trainable parameters. However, this model's performance is less compared to other state-of-the-art models

### Local binary pattern (LBP)

The texture of leukemia cells is heterogeneous, which can be explored to categorize them. Hence, in the proposed work, we have used a powerful feature descriptor developed by Ojala et al.^52^. This descriptor associates the analysis of occurrences and local structure analysis by assigning binary patterns to each pixel ${p}_{c}$. After that, the difference between pixel ${p}_{c}$ grey level value and its circular region is evaluated with the radius R centred at ${p}_{c}$. The LBP of the central pixel ${p}_{c}$ is calculated as follows.

1 $$LBP_{Q,R} \left( {p_{c} } \right) = \mathop \sum \limits_{q = 0}^{Q - 1} (q_{c} - p_{c} )2^{q}$$

**Figure 2:**
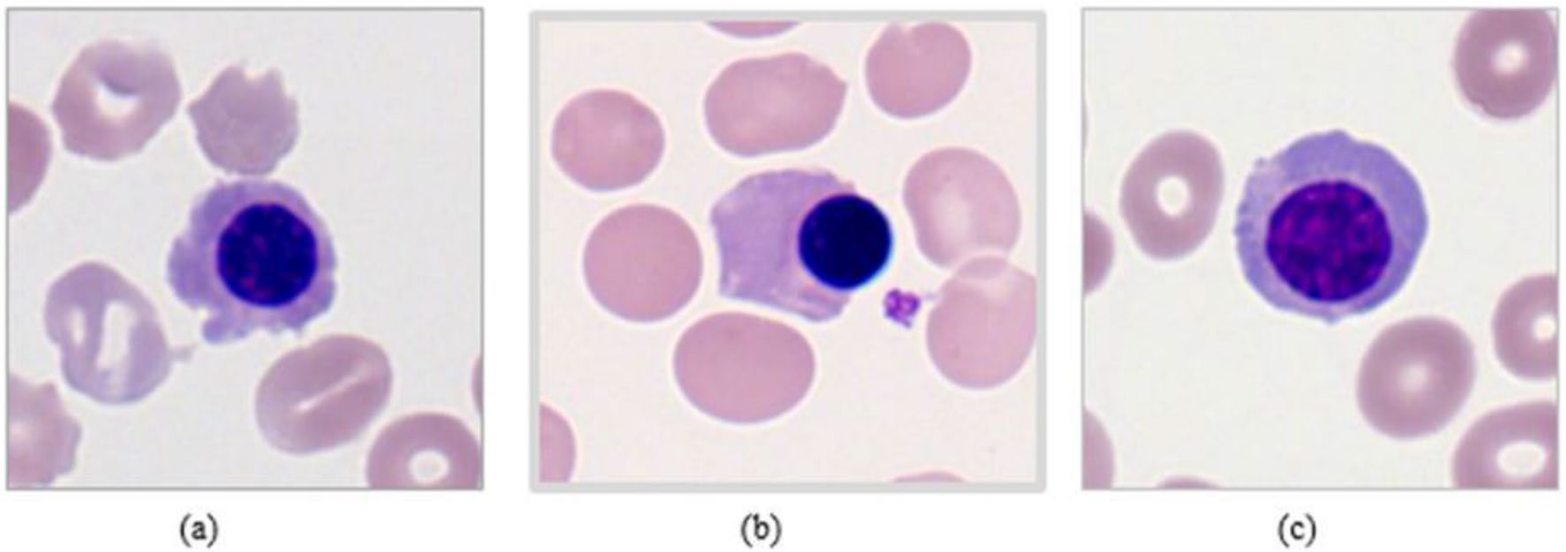
Here, (**a**–**c**) are sample images used in the experiment.

If the value of ${q}_{c}-{p}_{c}$>0, then 1 is assigned in the Eq. (1); otherwise, 0. Finally, the LBP picture is created by combining the texture descriptor and the LBP distribution pattern, as illustrated in Fig. 2. The histogram vector H of the LBP for image representation is given as follows.

2 $$H = \mathop \sum \limits_{i = 1}^{W} \mathop \sum \limits_{j = 1}^{D} \delta \left( {LBP_{Q,R} \left( {i,j} \right) - k} \right)$$

**Figure 3:**
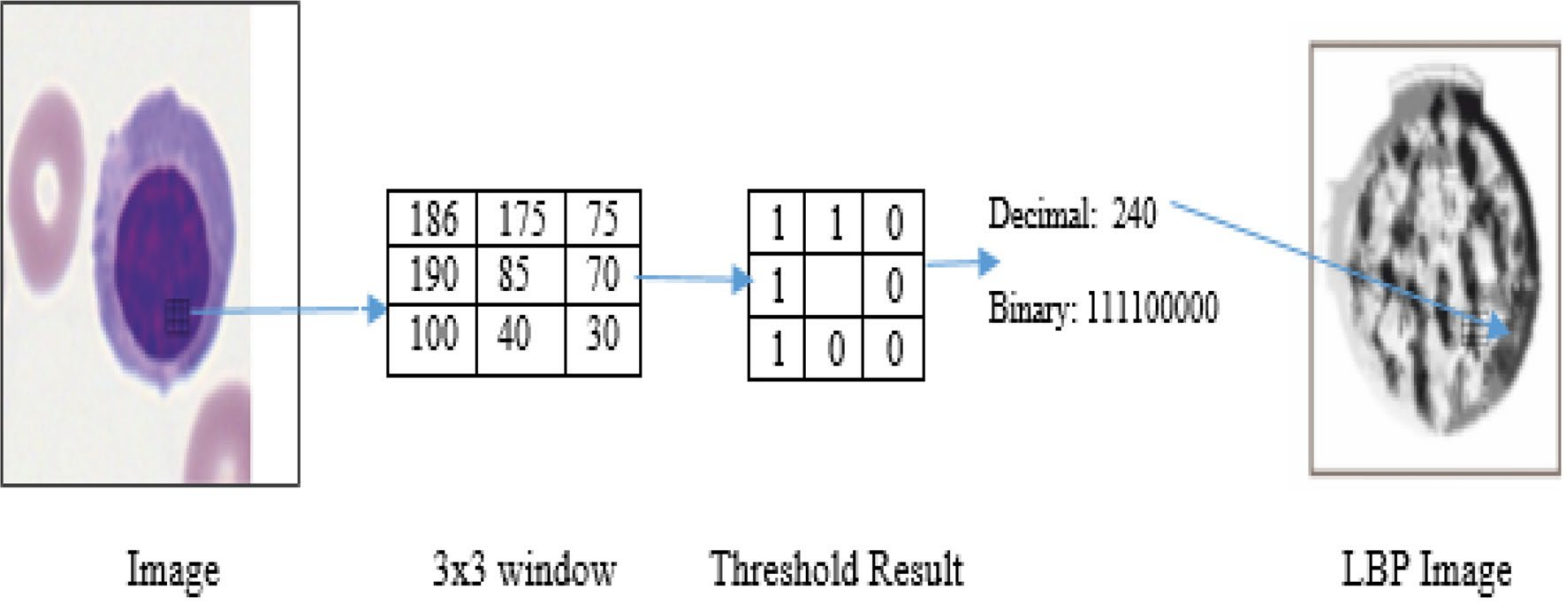
LBP image calculation.

**Figure 4:**
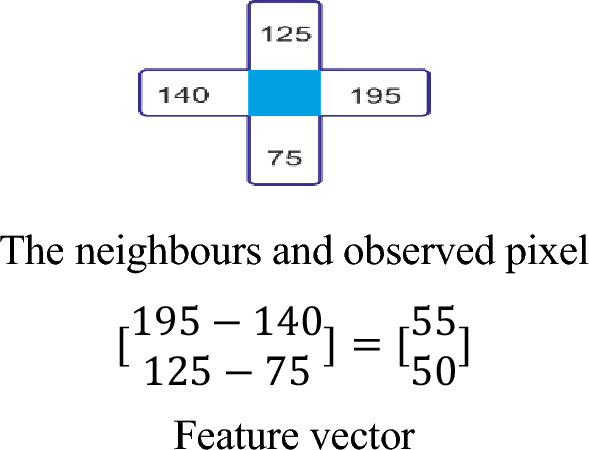
LBP image feature descriptor calculation.

The LBP image and their feature descriptor calculation are shown in Figs. 3 and 4, respectively.

### Histogram of oriented gradient (HOG)

Dalal and Triggs first used the HOG descriptor for object detection^53^. It focuses on the local shape and structure of an object. For the region of the image, the histogram is generated by calculating the magnitude and direction of the gradient. In the proposed work, images are resized to 256 × 256. After that, a sliding window of size 3 × 3 is used to calculate the gradient $Grad_{x}$ in the Y-direction and $Grad_{y}$ in the X-direction as follows.

3 $$Grad_{x} = Im(r,c + 1) - Im(r,c - 1)$$

4 $$Grad_{y} = Im(r - 1,c) - Im(r + 1,c)$$

where r and c refer to the row and column of the image. Finally, magnitude and direction are calculated using the following formulae.

5 $$Magnitude\left( M \right) = \sqrt {Grad_{x}^{2} + Grad_{y}^{2} }$$

6 $$Direction\left( D \right) = \arctan \left( {\frac{{Grad_{y} }}{{Grad_{x} }}} \right)$$

### Novel 3-scale deep CNN model (3SNet)

We have designed a novel 3-scale deep CNN model in the present study. The grey image and their corresponding LBP and HOG images are fed as input to the model. Each scale is seven layers deep and contains a convolution layer of 3 × 3 filter and Conv1, Conv2, Conv3, Conv4, Conv5 and Conv6 of sizes 16, 32, 64, 128, 256, and 512 respectively. After each convolution block, rectified linear unit (ReLU) and batch normalization (BN) are applied. The ReLU activation adds non-linearity to the model by applying a threshold to the pixels obtained from BN layers. This model has 9 × 10^9^ trainable parameters, and it can avoid degradation problems, saturation of the model and gradient descent problems^54^. The ReLU activation is defined as.

7 $$F\left( x \right) = \left\{ {\begin{array}{*{20}c} {0,x < 0} \\ {x,x > 0} \\ \end{array} } \right\}$$

where x = input to the layer. After each convolution layer, a max-pooling layer of size 3 × 3 and stride of 2 × 2 is incorporated. Finally, a global average pooling at each layer is applied that generates channel descriptors and combines them to develop feature fusion. The output from the fused feature acts as an input to a Fully Connected layer with 1024 filters followed by BN and ReLU activation. In the end, a dense layer of 15 neurons was added for AML-Cytomorphology_LMU respectively. The classification of multiclass classification is performed using the Softmax optimization function, which converts logits into probability. The input weight and bias calculate the probability value. Finally, the probability value is converted to a particular class of leukaemia cells. The value of the Softmax optimizer can be calculated using Eqs. (7) and (8).

8 $$P(x = k|\Phi^{\left( i \right)} ) = \frac{{e^{{\Phi^{\left( i \right)} }} }}{{\mathop \sum \nolimits_{k = 0}^{N} e^{{\Phi_{N}^{\left( i \right)} }} }}$$

9 $$\Phi = w_{0} y_{0} + w_{1} y_{1} + \ldots + w_{N} y_{N}$$

where N = 15, ${w}_{0}{y}_{0}$ = bias of kth class, $\Phi \, = \,$input vector, and the value of k = 0–14 for multiclass (15 class of leukemia cell).

### Feature fusion

Feature fusion improves the performance of the deep CNN. We have used three deep CNN models for feature extraction in the proposed method. The feature extracted from the HOG, Leukemia Cell and LBP image is fused as follows.

10 $$X = \left\{ {x_{1} ,x_{2} \ldots x_{n} } \right\}$$

11 $$Y = \left\{ {y_{1} ,y_{2} \ldots y_{n} } \right\}$$

12 $$Z = \left\{ {z_{1} ,z_{2} \ldots z_{n} } \right\}$$

**Figure 5:**
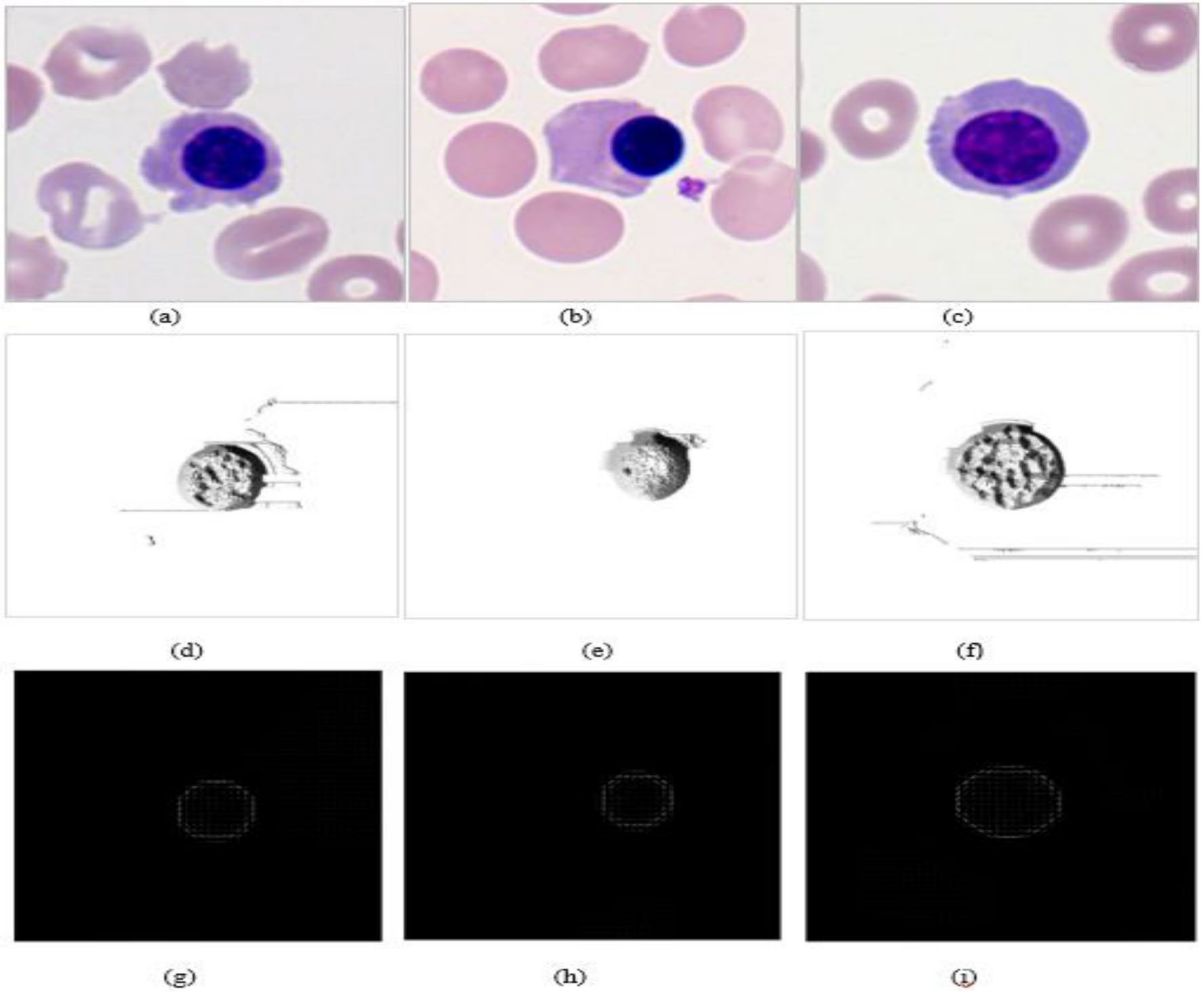
Here, (**a**–**c**) original image, (**d**–**i**) represents their corresponding LBP and HOG image respectively.

Respectively where, $n=512$. An enhanced features pool is generated by concatenations as follows.

13 $$F_{con} = X \oplus Y \oplus Z = \left( {x_{1} ,x_{2} , \ldots x_{n} ,y_{1} ,y_{2} , \ldots y_{n} ,z_{1} ,z_{2} , \ldots z_{n} } \right)$$

where ${F}_{con}$ is final feature vector with a bag of 1536 features. The original image and their LBP and HOG image is shown in Fig. 5.

#### .


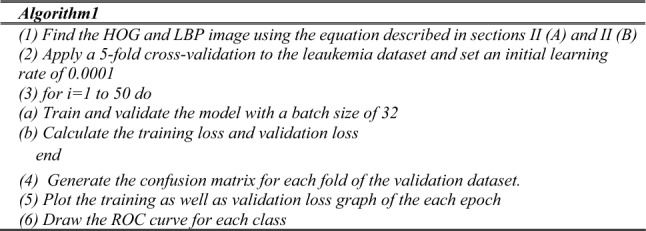


### Consent to participate

The authors declare their consent to participate in this article.

## Results

### Dataset

The images used in this research have been taken from the available Munich AML Morphology Dataset, containing 18,365 expert-labelled single-cell images^55^. These single-cell images were produced using the M8 digital microscope/scanner from peripheral blood smears of 100 people from each group, with the first group comprised of patients diagnosed with Acute Myeloid Leukemia at Munich University Hospital between 2014 and 2017and the second group having patients without signs of hematological malignancy.

### Training and validation

**Figure 6:**
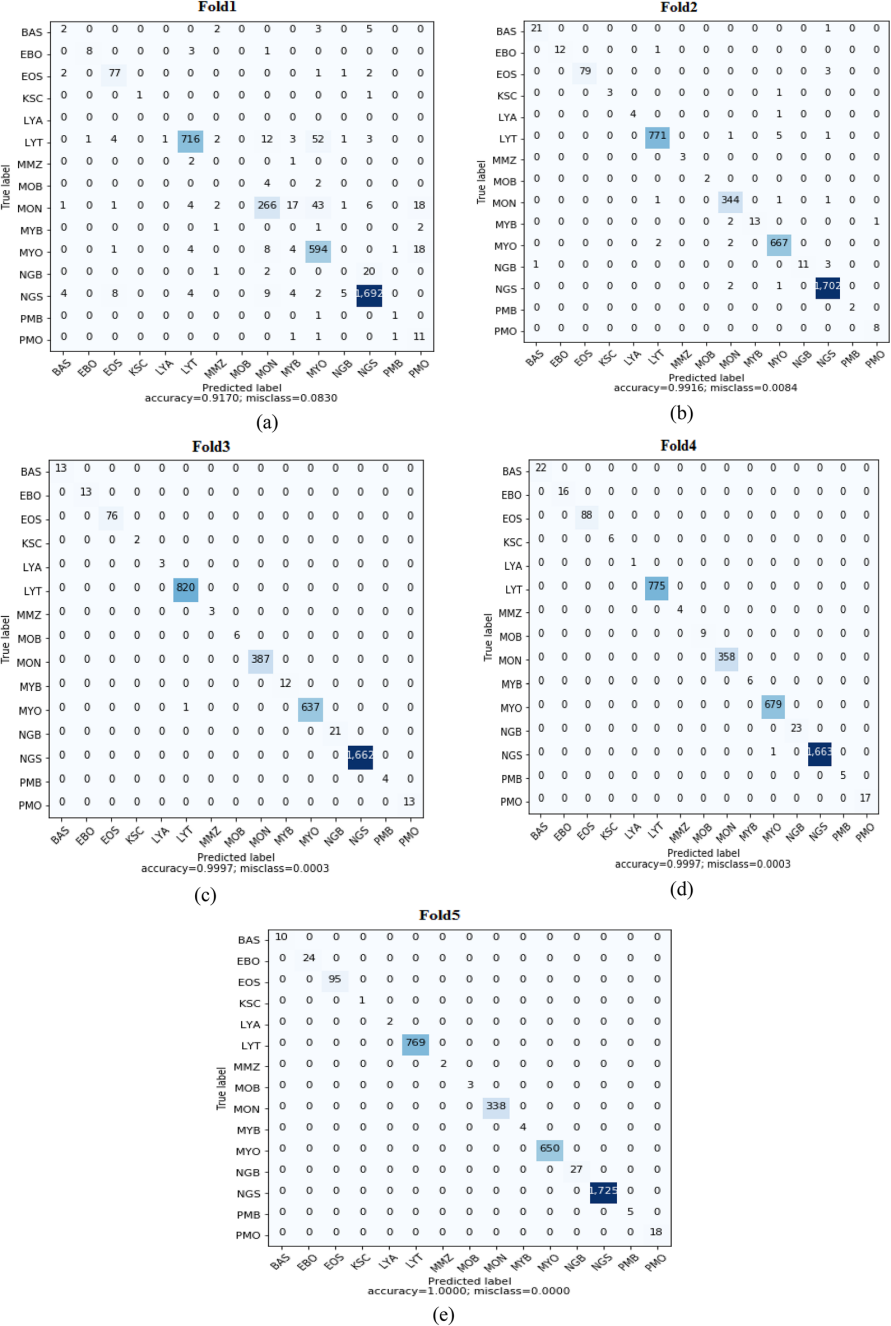
The confusion matrix of fold1, fold2, fold3, fold4 and fold5 are shown in the (**a**–**e**) respectively.

The training and validation of the proposed method are performed in Python 3.6, Tensorflow 2.0, Windows 10, Nvidia GeForce GTX TITAN X GPU with 128 GB RAM. The leukemia cells like lymphocyte and Promyelocyte have very similar morphological characteristics. Also in the dataset few classes like Lymphocyte, Basophil, Promyelocyte, Promyelocyte (bilobed), Myelocyte, Metamyelocyte, Monoblast, Erythroblast, and Smudge cells have less than 100 images. Due to this high classification, accuracy is difficult to achieve. Considering these challenges, a multimodal features fusion-based model has been proposed to discriminate 15 classes of leukemia cells. The 3SNet model is trained with an image size of 256 × 256 pixels and batch size 32 for 50 epochs. The initial learning rate was set to 0.0001. Since the dataset is imbalanced, we have applied fivefold cross-validation to avoid the biased performance of the model. In a fivefold cross-validation for each fold, one set is used for validation and four sets are used for training. Hence, in each fold, 20% images are used for validation and 80%, of images are used for training. In Fig. 6, we have depicted the confusion matrix of each fold. From the confusion matrix average performance measures like precision, recall, F1-score and accuracy are calculated.

**Figure 7:**
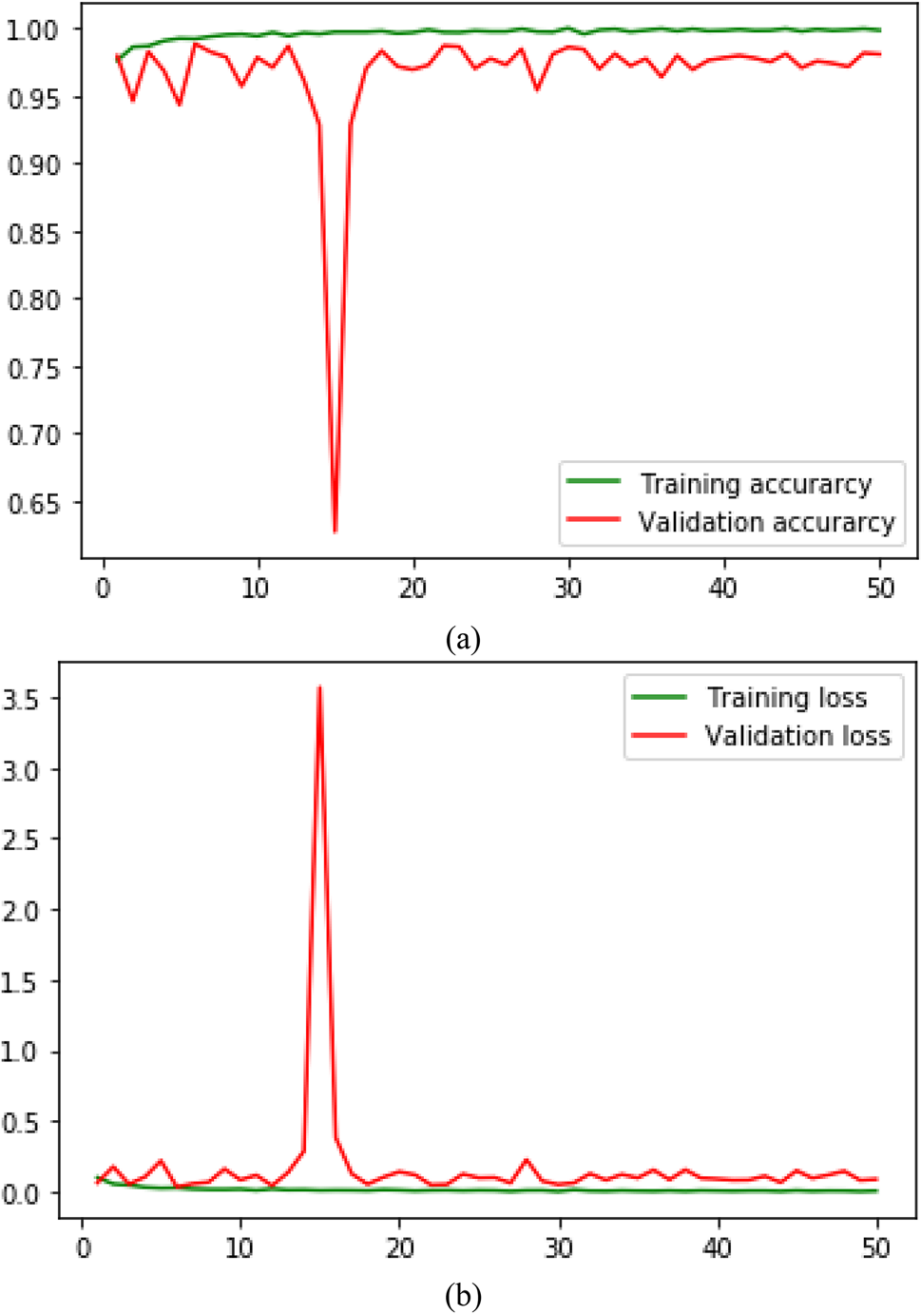
The training and Validation accuracy and loss of 3SNet is shown in (**a**) and (**b**) Respectively.

The loss function categorical_crossentropy is used to calculated the training and validation loss of the proposed method and shown in the Fig. 7. We can see in Fig. 7a that initially, validation accuracy fluctuates, but after 40 epochs, changes are negligible. Similarly, in Fig. 7b, training loss reaches close to zero. In addition, initially, validation loss fluctuates and becomes less vibrant after 40 epochs. This shows that the 3SNet model can differentiate leukemia cells with high accuracy and less training and validation loss.

The performance measures of the model are calculated for each fold, as shown in Table 3. Table 3, shows precision, recall F1-score, and accuracy values for each fold. It can be observed, in fold-1, that model performance is less than 50%. After that, it gradually increases in substituent folds. Finally, we can see proposed model achieved an average of 87.93% precision, 88.65% recall, 88.11% F1-score, and 98.16% accuracy.

**Table 3:** The performance measures of the 3SNet model.

Folds	Performance measures			
	Precision (%)	Recall (%)	F1-Score (%)	Accuracy (%)
Fold1	47.03	44.66	45.34	91.70
Fold2	92.60	98.60	95.26	99.16
Fold3	100	100	99.98	99.97
Fold4	100	100	99.99	99.97
Fold5	100	100	100	100
Average	87.93	88.65	88.11	98.16

## Discussion

Microscopic image analysis for blood smear provide essential data for diagnosing and predicting diseases in hematological assessment. Blood comprises three major components red blood cells (RBCs), white blood cells (WBCs) and platelets. Out of these, white blood cells (WBCs) are a part of the immune system and play an important role in the body’s immune system. Leukemia, a blood malignancy that affects the bone marrow and lymphatic system, is generally caused by abnormalities in these WBCs. The morphological differences in the lymphocytes in blood and bone marrow from patients with chronic lymphocytic leukemia and healthy ones have been noticed in various studies. These morphological differences can potentially diagnose the malignancy at various stages, from the primary to the acute stage. Nevertheless, the manual detection of these morphological differences needs expertise, effort and time. Due to this, it is very difficult to identify these cells, and it is necessary to automate this diagnosis with the help of CNN. In this study, we have used a dataset of 18,365 leukemia cells divided into 15 classes. The expert annotates the dataset, which is unbalanced due to the unequal distribution of data. In addition, out of 15 classes, nine classes contain less than 100 images. In Table 4 we have presented a summary several methods using different CNN models on different datasets.

**Table 4:** Comparison of 3SNet with the recent deep learning methods.

Study	Deep learning model	Images in the dataset	Accuracy (Leukemia detection)	Leukemia subtype accuracy
Thahn et al.^35^	CNN	108	96.6%	NR
Shafique et al.^56^	(AlexNet)	260	99.5%	96.06%
Pansombut et al.^57^	CNN	363	81.74%	81.5% B-lymphoblasts
Ahmed et al.^58^	CNN	354	88.25%	NR
Jha et al.^59^	LDP	260	98.7%	NR
Prellberg et al.^60^	ResNeXt50	12,528	88.91%	NR
Matek et al.^34^	ResNeXt	18,365 (3,312 BL)	90%	94% myeloblasts 41% Ab. prom
Loey et al.^61^	AlexNet	564	100%	NR
Vogado et al.^62^	AlexNet,	377	99%	NR
Di Ruberto et al.^63^	AlexNet	33	94.1%	NR
Rehman et al.^64^	AlexNet	330	97.78%	NR
Huang et al.^65^	DenseNet121	1322	95.3%	95.25%
Boldú et al.^66^	VGG-16	16,450 (4825 BL)	94.2% (cell) 100% (smear)	89.5% (cell) 94.7% (smear)
Proposed 3SNet	CNN	18,365 (3312 BL)	98.16%	99% myeloblasts

In the past, several research on leukemia cells classification has been reported, shown in Table3. In this regard, Thahn et al.^35^ developed a CNN model for normal and abnormal cell classification. They applied the data augmentation technique to increase the dataset's size, and the model's classification accuracy is 96.6%. In a similar type of research, Shafique et al.^56^ classify blood smears and their three subtypes using AlexNet. The overfitting of the model is avoided using the data augmentation technique and achieves 96.06% classification accuracy. Pansombut et al.^57^ utilized machine and deep learning to classify leukemia cells. First, the feature is extracted using ConvNet; after that, the feature is optimized using a genetic algorithm and finally, a classification accuracy of 81.74% is obtained using a support vector machine (SVM). Ahmed et al.^59^ reported the comparative study of several machine-learning algorithms and the effect of data augmentation on training. They also proposed a deep CNN model for the classification of leukemia cells. Their model classifies leukemia cells with an accuracy of 88% and its subtype with an accuracy of 81%.

Prellber and Kramer et al.^60^ classify leukemia cells using ResNeXt50 with a Squeeze-and-Excitation block. They train their model with original and augmented images and archive a weighted F1-score of 89.91%. Many pieces of research on leukemia cell classification also applied a transfer learning-based approach. Loey et al.^61^ compare the performance of AlexNet before and after fine-tuning. They claim that fine-tuning AlexNet performed better and achieved an accuracy of 100%. In similar research, Vogado et al.^62^ applied three deep learning models AlexNet, Coffenet, and Vgg-f to extract features from the leukemia cells. In addition, two classifiers, SVM and KNN were applied for classification. They reported an SVM classifier to outperform and archived an accuracy of 99.76%. Ruberto et al.^63^ also extract features from pre-trained AlexNet. Nevertheless, before extracting features from leukemia cells, they applied preprocessing, detecting blob, and segmentation to extract objects of interest. Their method achieves 94.1% classification accuracy.

Rehman et al.^64^ extract features using the deep CNN model. Comparative analysis of three classifiers, Naive Base, KNN, and SVM, are performed using the deep features. Out of these three classifiers, Naïve Base achieved 78.34%, KNN 80.42%, SVM 90.91%, and proposed deep classifier 97.78%. Huang et al.^65^ also applied a transfer-learning approach to extract features from Leukemia cells. The Inception-V3, ResNet50, and DenseNet121 classify with a notable accuracy of 74.8%, 84.9% and 95.3% respectively.

In short, all these methods have a high potential for the classification of blood smears. However, many researchers experiment on small datasets, as data augmentation techniques have been used to increase the dataset size. Due to image augmentation, overfitting of the model can be avoided, but several images of the same type lead to the biased performance of the model. In addition, blood smears having a smaller number of images in the dataset need to be explored for their better classification. Therefore, we have not applied the data augmentation technique in the proposed method and focused on the blood smears having fewer images in the dataset. Features extracted from the HOG, Leukemia, and LBP images and aggregated together to form a feature fusion vector that improves the classification performance of the leukemia cell. The 3SNet is the three-scale sequential model used for feature extraction and classification. Each model is trained with the input of 256 × 256 pixels images with a batch size 32 for 50 epochs. Further, a fivefold cross-validation scheme is applied to the model to evaluate bias-free performance. The multi-scale fusion-based CNN model outperforms most blood smears, and outstanding performance is obtained for the cells with less than 100 images in the dataset. The average sensitivity and precision obtained from fivefold cross-validation for the cells with more than 1000 images in the dataset are more than 95%, while cells with less than 100 images in the dataset are 70%. The class-wise performance of each class cell has been compared with the method proposed by Matek et al.^34^.

Table 5 shows that the Neutrophil (segmented) cells have 8484 images, which is the highest number in the dataset. For the Neutrophil cell, the precision of the model is close to 99%, and the sensitivity is 99.4% better than the 96% of Matek et al.^34^. For other leukemia cells having more than 1000 images in the dataset, the fusion-based outperforms compared to the available method. Furthermore, the 3SNet is highly sensitive toward the cells having less than 100 images in the dataset. For such cells, except for the myelocyte cells, which had 76.2% precision, achieved more than 80% precision and 80% sensitivity. This notable precision and sensitivity confirm that the proposed 3SNet model can be used for real-time diagnosis.

**Table 5:** Class-wise performance of 3SNet and Matek et al.^34^ method.

Class	Sensitivity(proposed)/Matek et al.^34^	Precision (Proposed)/Matek et al.^34^	Images in the dataset
Mature leukocytes			
Neutrophil (segmented)	99.4/96	99.6/99	8,484
Neutrophil (band)	80/59	74.6/25	109
Lymphocyte (typical)	99.4/95	97.8/96	3,937
Lymphocyte (atypical)	80/7	76/20	11
Monocyte	97.2/90	94.6/90	1,789
Eosinophil	97/95	97.8/95	424
Basophil	83.40/82	82.4/48	79
Immature leukocytes			
Myeloblast	96.73/94	98.6/94	3,268
Promyelocyte	82.3/54	94.6/63	70
Promyelocyte (bilobed)	86.6/41	90/45	18
Myelocyte	80/43	76.2/46	42
Metamyelocyte	80/13	80/7	15
Monoblast	80/58	80/52	26
Erythroblast	97.80/87	91.8/75	78
Smudge cell	85/77	85/53	15
Total			18,365

**Figure 8:**
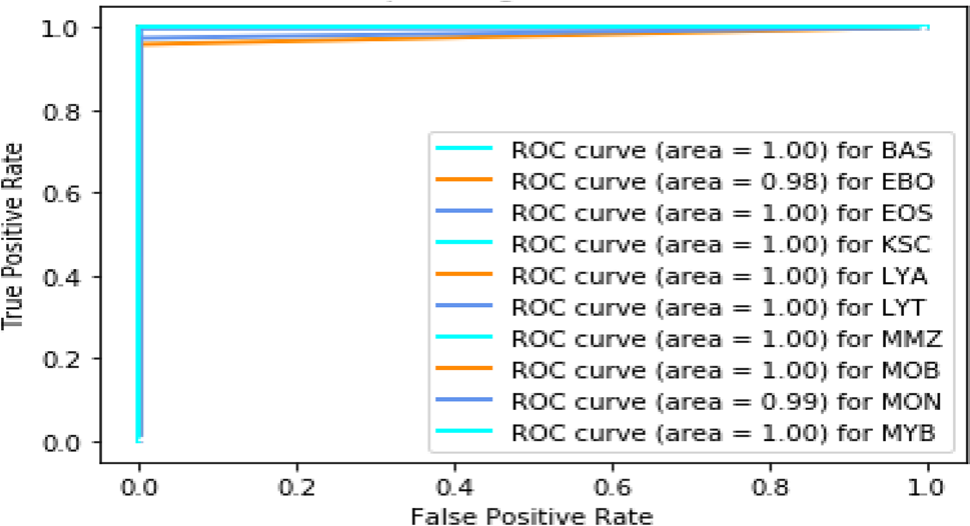
The ROC Plot for the proposed method.

**Figure 9:**
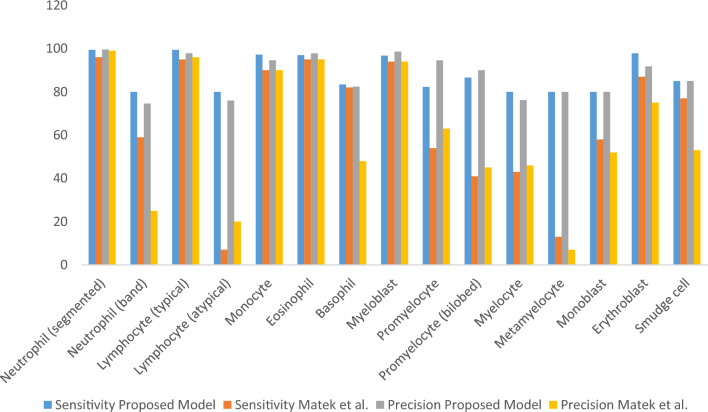
The bar plot for the comparison of precision and sensitivity with the method^34^.

Further, an receiver operating characteristic (ROC) curve is plotted for performance visualization, taking the true positive rate on the Y-axis and the false positive rate on the X-axis^67,68^, shown in Fig. 8. We can see in Fig. 8 that most of the leukemia cell ROC curve area is 1, while EBO shows 98% and MON 99%. This confirms that our model is highly sensitive towards leukemia identification. The class-wise performance can also be observed using the bar chart shown in Fig. 9. We can see in Fig. 9 that the proposed 3SNet model sensitivity and specificity are better than the state-of-the-art method.

### Ablation study of the proposed model

We conducted two experiments on similar settings, as discussed in "Training and validation" section. However, we changed the setting of the proposed model as follows: In the first experiment, we removed the HOG feature and trained the model for 50 epochs in a batch size of 32. After training of the model, performance measures precision, recall, F1-score and accuracy of the model are calculated as shown in Table 6. Table 6 shows that the 3SNet achieved average precision and F1-score of 86.60% and 85.10%, respectively.

**Table 6:** The performance measures of the 3SNet mode using Grey and LBP features.

Folds	Performance measures			
	Accuracy (%)	Precision (%)	Recall (%)	F1-Score (%)
Fold1	88.27	46.15	41.83	43.88
Fold2	96.49	90.48	91.72	91.09
Fold3	97.06	98.17	94.28	96.18
Fold4	97.67	98.87	95.19	96.30
Fold5	98.73	99.35	96.81	98.06
Average	95.64	86.60	83.96	85.10

In the second experiment, we removed the LBP feature, and the model was trained using gray and HOG features for 50 epochs in a batch size of 32. The average performance measures are shown in Table 7. In Table 7, we can observe that the model achieved an accuracy of 96.13% and a recall value of 84.61%.

**Table 7:** The performance measures of the 3SNet mode using Grey and HOG features.

Folds	Performance measures			
	Accuracy (%)	Precision (%)	Recall (%)	F1-Score (%)
Fold1	87.15	45.04	42.74	43.85
Fold2	97.23	89.87	93.18	91.50
Fold3	98.42	97.28	92.59	94.87
Fold4	98.78	99.51	96.94	98.21
Fold5	99.11	99.76	97.64	98.69
Average	96.13	86.29	84.61	85.42

The dataset used in the study is divided into training and validation. The proposed method applied a similar training and validation set as utilized by Matek et al.^34^. However, we conducted an ablation study and divided the dataset into 80%, 10%, and 10% for training, validation and testing, respectively. The class-wise sensitivity and precision of each cell on the test dataset are shown in Table 8. In Table 8, we notice that the sensitivity and precision of the cells with large numbers of images is more than 90%. Furthermore, the cells having fewer images also achieved notable performance measure values.

**Table 8:** Class-wise performance of the proposed 3SNet on the test dataset.

Class	Sensitivity	Precision	Images in the dataset
Mature leukocytes			
Neutrophil (segmented)	96.53	97.27	8484
Neutrophil (band)	75.16	68.94	109
Lymphocyte (typical)	94.32	91.50	3937
Lymphocyte (atypical)	58.46	42.37	11
Monocyte	94.81	90.12	1789
Eosinophil	95.76	93.48	424
Basophil	80.39	78.21	79
Immature leukocytes			
Myeloblast	92.75	96.34	3268
Promyelocyte	79.18	81.56	70
Promyelocyte (bilobed)	73.67	76.19	18
Myelocyte	76.84	72.30	42
Metamyelocyte	52.23	57.82	15
Monoblast	76.14	78.75	26
Erythroblast	94.59	86.60	78
Smudge cell	55.27	48.58	15
Total			18,365

## Conclusion

This research proposes a novel 3SNet, a deep CNN model for leukemia cell classification. Leukemia cells are a major cause of blood cancer. These blood smears' morphological characteristics are very similar in several classes. Due to this, classification tasks are difficult. To tackle this problem, our method implicitly extracts features from leukemia and their corresponding HOG and LBP images using 3SNet. The HOG feature locates the local shape, and the LBP feature describes the texture pattern of leukemia cells, which helps to discriminate the morphological characteristics of blood smears. The features extracted from three scales are fused and refined to enhance the feature pool. After that, the feature vector is passed to the classification module. The classification performance depicted in Table 5, confirms that the proposed method not only classifies cells having a large number in the dataset with high accuracy but also cells having a smaller number of images in the dataset. Further, depth-wise separable convolution block reduces the computation cost and resources. Hence, this method can be used to design computer-aided diagnostic (CAD) tools that can provide a second opinion to a doctor. The limitation of the model is to feed the images at three scales for training. In addition, the computation costs of the algorithm can be further reduced. In future work, we will add other texture features and a grayscale image to the deep CNN model for further performance improvement. In addition, feature optimization techniques can be applied to the feature pool to enhance the fused features. Further, other lightweight deep CNN models with attention mechanisms can be explored to improve the classification performance. The 2D convolutional layers of the proposed model can be replaced with 3D convolution layers to perform analysis of the 3D images. This will improve the model's capability to diagnose disease more accurately.

## Data Availability

The data supporting this study’s findings are available from the corresponding author upon reasonable request.
